# Sensitivity of lateral flow technique for diagnosis of canine parvovirus

**DOI:** 10.1038/s41598-024-55548-x

**Published:** 2024-03-01

**Authors:** M. S. Abousenna, R. H. Sayed, Shaimaa A. E., F. A. Shasha, Sara E.A El Sawy, D. M. Darwish

**Affiliations:** Central Laboratory for Evaluation of Veterinary Biologics, Agricultural Research Center, P.O. Box 131, Cairo, 11381 Egypt

**Keywords:** CPV, Canine parvovirus vaccine, Antigen testing, Diagnostic testing, Gold nanoparticles, Immuno-chromatographic, Lateral flow assay, Sensitivity and specificity, Microbiology, Virology, Biosensors

## Abstract

In this study, we devised a nanogold lateral flow immunoassay (LFA-CPV antigen test) for detecting canine parvovirus (CPV) in living attenuated CPV vaccines. We conducted instrumental characterization of the prepared nanogold particles and the developed LFA-CPV antigen test was rigorously evaluated for its performance verification including limit of detection, sensitivity, specificity, selectivity and accuracy. The LFA-CPV antigen test demonstrated strong performance when assessed against qPCR using different batches of live attenuated CPV vaccines, indicated a sensitivity of 96.4%, specificity of 88.2%, and an overall accuracy of 95%. These results suggest that the developed LFA-CPV antigen test could serve as a viable alternative for evaluation live attenuated CPV vaccines, and provide it as a point of care test for CPV diagnosis, offering a potential substitute for traditional laboratory methods, particularly qPCR.

## Introduction

Canine Parvovirus (CPV) infection is a major cause of severe gastroenteritis in domestic and wild canids worldwide, significantly impacting their health and survival^1,2^. This diminutive, non-enveloped virus, classified within the Parvoviridae family, harbors a single-stranded DNA genome spanning approximately 5.2 kilobases. Recognized as a significant threat, Canine Parvovirus (CPV) has emerged as a leading cause of illness, resulting in pronounced symptoms including severe vomiting, bloody diarrhea, dehydration, and an elevated risk of mortality, especially in unvaccinated dogs and young puppies^3,4^. The diagnostic process for CPV hinges on a range of laboratory tests, notably virus isolation, polymerase chain reaction (PCR), and enzyme-linked immunosorbent assay (ELISA). Nevertheless, these diagnostic methods come with inherent challenges, including the necessity for sophisticated equipment, specialized laboratory settings, well-trained personnel, as well as being time-consuming and financially burdensome^5^. Conventional methods like virus isolation and electron microscopy are hampered by their time-consuming nature, limited sensitivity, and high cost^6,7^. Early and accurate diagnosis of parvovirus infection is paramount to controlling the disease, on the other hand, Canine parvovirus vaccines harboring live attenuated virus can be utilized as clinical samples for diagnostic assessment. Direct canine parvovirus antigen-based techniques like LFAs offer an alternative to qPCR, potentially representing the sole viable, rapid, and cost-effective solution for resource-limited settings.

The lateral flow assay (LFA) emerges as a game-changer in diagnostics, offering a rapid, user-friendly, and cost-effective approach for detecting various infectious diseases in humans and veterinary medicine^8,9^. Utilizing gold nanoparticles linked to specific antibodies, LFA identifies viral antigens in samples, producing visible bands on a test strip. However, the sensitivity and specificity of LFA can vary based on factors such as the targeted antigenic protein, assay format, and the quality of reagents employed^9,10^.

The performance of LFA for CPV diagnosis remains a subject of ongoing investigation, with studies yielding inconsistent results. While some reports demonstrate high sensitivity and specificity compared to conventional methods^11,12^. Conversely, other studies have identified lower sensitivity, especially in samples exhibiting minimal viral loads^13,14^. Moreover, the performance of LFA is susceptible to influencing factors like the sample type, storage conditions, and the duration between sample collection and testing^15^.

The Central Laboratory for Evaluation of Veterinary Biologics (CLEVB) systematically assesses live attenuated Canine Parvovirus (CPV) vaccine batches, focusing on sterility, identity, safety, and potency. While sterility and safety evaluations are straightforward, the identity and potency assessments have traditionally employed time-consuming and costly methods, such as quantitative Polymerase Chain Reaction (qPCR) and tissue culture virus titration using an Indirect Fluorescent Antibody (IFA) test. However, the identification process has posed challenges, leading to the rejection of numerous CPV vaccine batches due to identity issues. To streamline and expedite this crucial aspect of the evaluation, CLEVB is actively exploring alternative methods that maintain accuracy while reducing costs and processing time. This initiative aims to enhance efficiency in CPV vaccine assessment and ensure the timely approval of batches meeting stringent quality standards.Therefore, the aim of this study was to evaluate the sensitivity of LFA for the detection of CPV2 using a panel of well-characterized live attenuated CPV vaccine batches and compare it to the qPCR.

## Materials and methods

### Canine parvovirus vaccines

Various types of Canine parvovirus vaccine batches (n = 100) were supplied by the central laboratory for evaluation of veterinary biologics (CLEVB), Cairo, Egypt. The vaccines are monovalent, bivalent and polyvalent either locally manufactured or imported. These batches (n = 100) containing modified live attenuated CPV2 virus which provide cross protection against CPV2 mutants (CPV2a, CPV2b, and CPV2c)^16,17^ Table S1 (supplementary information) and had been selected for this study and were previously evaluated with satisfactory and unsatisfactory results by CLEVB.

Monovalent live attenuated CPV vaccine (n = 1) was tested previously by qPCR, tissue culture virus titration using IFA test, and kept as reference (10^6^ TCID_50_/0.1 ml), it was used for assessment the limit of detection (LOD) of the LFA-CPV antigen test.

The selection of various types of Canine Parvovirus (CPV) vaccine batches (n = 100) for the study conducted by the Central Laboratory for Evaluation of Veterinary Biologics (CLEVB) was a deliberate and rationale-driven process. These batches, comprising monovalent, bivalent, and polyvalent vaccines, were chosen based on specific criteria to ensure the diversity and representativeness of the study sample.

The rationale behind selecting these batches includes the following key points:

1. Monovalent, Bivalent, and Polyvalent Variants: The inclusion of monovalent, bivalent, and polyvalent vaccines allowed for a comprehensive assessment of different vaccine formulations. This diversity is essential for understanding the performance of the LFA-CPV antigen test across various vaccine types.
2. Local and Imported Variants: Both locally manufactured and imported vaccine batches were included, providing a broader perspective on vaccine sources. This accounts for potential variations in manufacturing processes, quality control, and the origin of the vaccines.
3. Cross Protection Against CPV2 Mutants: The selected batches contained modified live attenuated CPV2 virus, providing cross-protection against CPV2 mutants (CPV2a, CPV2b, and CPV2c). This aspect was crucial to evaluating the vaccines' effectiveness in addressing a range of CPV variants.
4. Previous Evaluation by CLEVB: The batches had undergone prior evaluation by CLEVB, yielding both satisfactory and unsatisfactory results. This background information contributes to a comprehensive understanding of the vaccines' performance based on established quality and safety standards.
5. Reference Monovalent Vaccine for LOD Assessment: A monovalent live attenuated CPV vaccine (n = 1) was included as a reference with a known titer (106 TCID50/0.1 ml). This specific vaccine served as a benchmark for assessing the Limit of Detection (LOD) of the LFA-CPV antigen test.

In summary, the selection of these vaccine batches was not arbitrary but driven by a strategic rationale to encompass a diverse range of formulations, sources, and protective capabilities. The inclusion of both locally produced and imported vaccines, along with the assessment of LOD using a reference monovalent vaccine, enhances the study's comprehensiveness and relevance to real-world scenario.

### Monoclonal antibodies against CPV2 VP2 protein

Monoclonal antibodies (MAbs) with specificity to both recombinant Canine Parvovirus 2 (CPV-2) capsid protein VP2 and CPV from clinical samples (Cat.# 3PV16) were procured from HyTest Ltd, located in Finland. These antibodies were obtained through a process involving the derivation of hybridoma clones. The hybridomas were created through the fusion of Sp2/0 myeloma cells with spleen cells isolated from Balb/c mice that had been immunized with canine parvovirus. This hybridization process resulted in the generation of monoclonal antibodies that exhibit specificity to the CPV-2 capsid protein VP2, as well as CPV present in clinical samples.

### Goat anti-mouse IgG

Goat anti-Mouse IgG Fc secondary antibody (Cat# 31168) was purchased from Thermo Fisher Scientific. USA.

### Preparation of polyclonal antibodies against CPV in rabbits^9^

The monovalent vaccine for Canine Parvovirus was combined with a volume/volume percent solution of complete Freund's adjuvant. A total of twenty-five male rabbits were administered intradermal inoculations of the emulsion at a dosage of 0.1 mg per dose. Subsequently, booster doses of the Canine Parvovirus monovalent vaccine, along with an incomplete Freund's adjuvant mixture, were administered subcutaneously at 0.1 mg per dose to the previously immunized rabbits at intervals of the 2nd, 4th, 6th, and 8th weeks.

Serum samples were collected at 5 days following the last inoculation and subjected to testing for CPV-specific antibodies using an agar gel precipitation test. If the serum sample contains antibodies to CPV antigens, they bind together, forming a complex of interlaced antigen–antibody, which then precipitates in the agar. The precipitation can be observed with the naked eye as a thin white line.

### Purification of immunoglobulin of rabbit antibodies using caprylic acid^18^

The serum samples collected from rabbits immunized with the Canine Parvovirus monovalent vaccine were combined and subjected to centrifugation at 12,000 rpm for 30 min. The resulting pellet was discarded. The collected serum was then diluted 1:3 using sodium acetate buffer (0.06 M, pH 4.6) in a beaker equipped with a magnetic stir bar. The mixture was stirred using the magnetic bar.

After 30 min of stirring at room temperature, 2 ml of caprylic acid was added dropwise to the diluted rabbit serum. The mixture was then centrifuged at 12,000 rpm for 30 min. The supernatant was carefully collected, and the dialysis process was initiated. The supernatant was dialyzed overnight against PBS buffer at 4 °C. During the dialysis process, two or three buffer changes were performed to remove any excess caprylic acid.

Finally, the purified immunoglobulin concentration was determined using a spectrophotometer.

### Preparation of nanogold particles of 20 nm diameter size^9,19^

To formulate the gold nanoparticles, a process was carried out as follows:

First, 50 ml of ultrapure water was added to a boil with vigorous stirring using a hot plate stirrer. During this process, sodium citrate at a concentration of 0.01% (w/v) was added to the boiling water.

Next, 1 ml of HAuCl4 solution at a concentration of 1% was added to the solution. As the solution underwent the reaction, its color changed to red, indicating the formation of gold nanoparticles.

Subsequently, sodium azide at a concentration of 0.02% (w/v) was utilized in the solution. The solution was then allowed to cool.

### Instrumental characterization of prepared nanogold particles

The UV–Vis absorption spectra of the synthesized gold nanoparticles (Au NPs) were determined using a UV–Vis spectrophotometer (Shimadzu UV-3600, Japan) across the wavelength range of 200 to 800 nm.

X-ray diffraction (XRD) and selected area electron diffraction (SAED) patterns of the Au NPs were recorded in reflection mode at a scan rate of 0.04 degrees per second. This was achieved using a Shimadzu LabX-XRD-6000 instrument with CuKα radiation (λ = 1.5406 Å). The XRD system was operated at 30 kV voltage and 30 mA current within the 2θ range of 5o–60°. The XRD peaks were indexed using the X’pert High Score software program.

Interior morphology and structural verification of gold were investigated using high-resolution transmission electron microscopy (HR-TEM) with a JEOL JEM 1400 instrument in Tokyo, Japan. The HR-TEM was operated at 15 kV, and elemental composition was determined through energy-dispersive X-ray spectroscopy (EDX) analysis.

### Conjugation of nanogold particles with monoclonal antibodies against CPV VP2 protein^20^

The pH of the nanogold particles was adjusted to 8.5 using a 0.02 M K_2_CO_3_ solution. With gentle stirring, 1 ml of monoclonal antibodies against the CPV VP2 protein at a concentration of 1 mg/ml was mixed with 100 ml of the prepared nanogold particles. The mixture was lightly shaken for 15 min.

To block any unreacted sites, 1% m/v polyethylene glycol (PEG-20000) was added to the mixture with gentle stirring for another 15 min. Afterward, the mixture was subjected to centrifugation at 12,000 rpm for one hour.

The resulting conjugated nanogold particles were then suspended in 1 ml of dilution buffer containing 3% (w/v) sucrose, 20 mM Tris, 1% (w/v) bovine serum albumin, and 0.02% (w/v) sodium azide. The suspension was stored at 4 °C for further use.

### Dispensing of conjugated nanogold particles with monoclonal antibodies against CPV VP2 protein, nonconjugated CPV-specific rabbit IgG, and goat anti-mouse IgG on nitrocellulose membrane and conjugation pad^21^

*The sample pad*: consisted of glass fiber (Ahlstrom 222), which was pretreated with a buffer solution at pH 8.5. The buffer solution was prepared using ultrapure water and contained the following components: 1% (w/v) PVP, 2% (w/v) Titron X100, 3.81% (w/v) Borax, 0.1% (w/v) casein sodium salt, 0.15% (w/v) SDS, 0.5% (w/v) sodium cholate, and 0.02% (w/v) sodium azide.

After pretreatment, the sample pad was dried at 37 °C to remove any remaining moisture.

*The conjugation pad*: glass fiber (Ahlstrom 8964): was pretreated with a conjugation treated buffer solution at pH 7.4. The buffer solution consisted of 20 mM PBS containing 2% (w/v) BSA, 2.5% (w/v) sucrose, 0.3% (w/v) PVP, 1% (w/v) Triton × 100, and 0.02% (w/v) sodium azide. After pretreatment, the conjugation pad was dried at 37 °C to remove any remaining moisture.

Next, the pretreated conjugation pad was saturated with monoclonal antibodies against CPV VP2 protein conjugated nanogold particles. It was then dried at 37 °C for one hour and kept in a dry condition for further use.

*Nitrocellulose (NC) membrane (mdi CNPF-PD31)*: using a dispenser (Iso-flow), two lines were dispensed onto the NC membrane measuring 300 mm × 25 mm. The test line was dispensed with purified rabbit IgG specific to CPV (1.2 mg/1 ml) at a volume of 1 µl per 1 cm line. The control line was dispensed with goat anti-mouse IgG (0.6 mg/ml) at a volume of 1 µl per 1 cm line.

After the dispensing process, the loaded NC membrane was dried at 37 °C for four hours and then kept in a dry condition.

**Figure 1:**
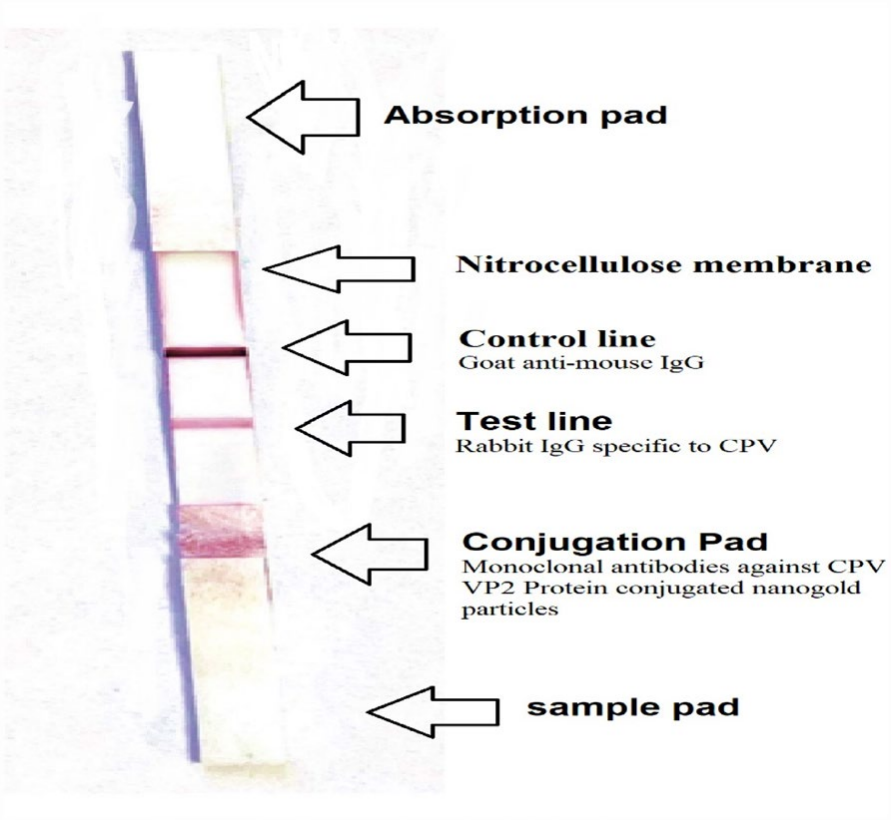
The description of the lateral flow assay (LFA) using the prepared LFA strip reveals the inclusion of distinct components: a sample pad, conjugation pad, nitrocellulose membrane, test line, control line, and an absorption pad.

To assemble the components, a PVC card was used to adhere the treated sample pad, conjugated pad, loaded NC membrane, and absorbent pad together. The assembled structure was then cut into 4 mm width as shown in Fig. 1.

### Analytical specificity testing

The LFA-CPV antigen test was tested with different viral strains, including Canine adenovirus, Canine Parainfluenza, and Canine distmper. Table S2 (supplementary information). Each strain was tested at a titer of 10^6^ TCID_50_/mL.

The LFA- CPV antigen test was also tested for cross-reactivity with different bacterial strains (10^7^ CFU/0.1 mL), including *Escherichia coli*, *Staphylococcus aureus*, *Salmonella Typhimurium*, and *Streptococcus pyogenes*. In addition, the test was tested for reactivity with interfering substances, such as whole blood, dexamethasone, and phenylephrine.

### Analytical sensitivity testing of the developed LFA-CPV antigen

The limit of detection of the LFA-CPV antigen test was determined by testing tenfold serial dilutions of the virus starting from 10^6^ TCID_50_/0.1 mL to 10 TCID_50_/0.1 mL, This assessment involved both the developed LFA-CPV antigen test and qPCR following the methodology outlined by Decaro et al.^22^. The primers and probe are detailed in Table S3 (the supplementary information), with a ct value threshold equal or exceeding 37 indicating a negative outcome.

### Determination of the sensitivity, specificity, and accuracy of the developed LFA-CPV antigen compared with the qPCR test

The sensitivity, specificity, and accuracy of the LFA-CPV antigen test were determined by comparing it to qPCR test. One hundred cainine parvovirus vaccine batches were tested with both the LFA-CPV antigen test and qPCR test. The samples were considered positive if qPCR indicated a positive result, while they were considered negative if qPCR indicated a negative result^8,23^.

### Ethical approval for animal experiments

The current study is reported in accordance with the Animal Research: Reporting of In-Vivo Experiments (The ARRIVE guidelines 2.0). All procedures involving animal use throughout the study strictly adhered to the guidelines set by the Institutional Animal Care and Use Committee at the Central Laboratory for Evaluation of Veterinary Biologics. Approval for this study was obtained from the ethical committee of the Institutional Animal Care and Use Committee at the Central Laboratory for Evaluation of Veterinary Biologics (Related information). The manuscript is considered compliant with bioethical standards in good faith.

No anesthesia or euthanasia protocols were employed for the animals involved in this study, as all animal-dependent methodological procedures were categorized as either no or low-pain procedures that can be ethically performed on a conscious and alive animal.

### Statistical analysis

The data underwent comprehensive analysis using IBM SPSS version 21 for Windows (SPSS Inc., Chicago, IL, USA). Within this analytical framework, confidence intervals were meticulously calculated for both the qPCR and the developed LFA-CPV antigen test. This robust statistical approach provided a nuanced understanding of the precision associated with the measurements from these two diagnostic methods.

## Results

### Gold NPs stutcture verification^24^

**Figure 2:**
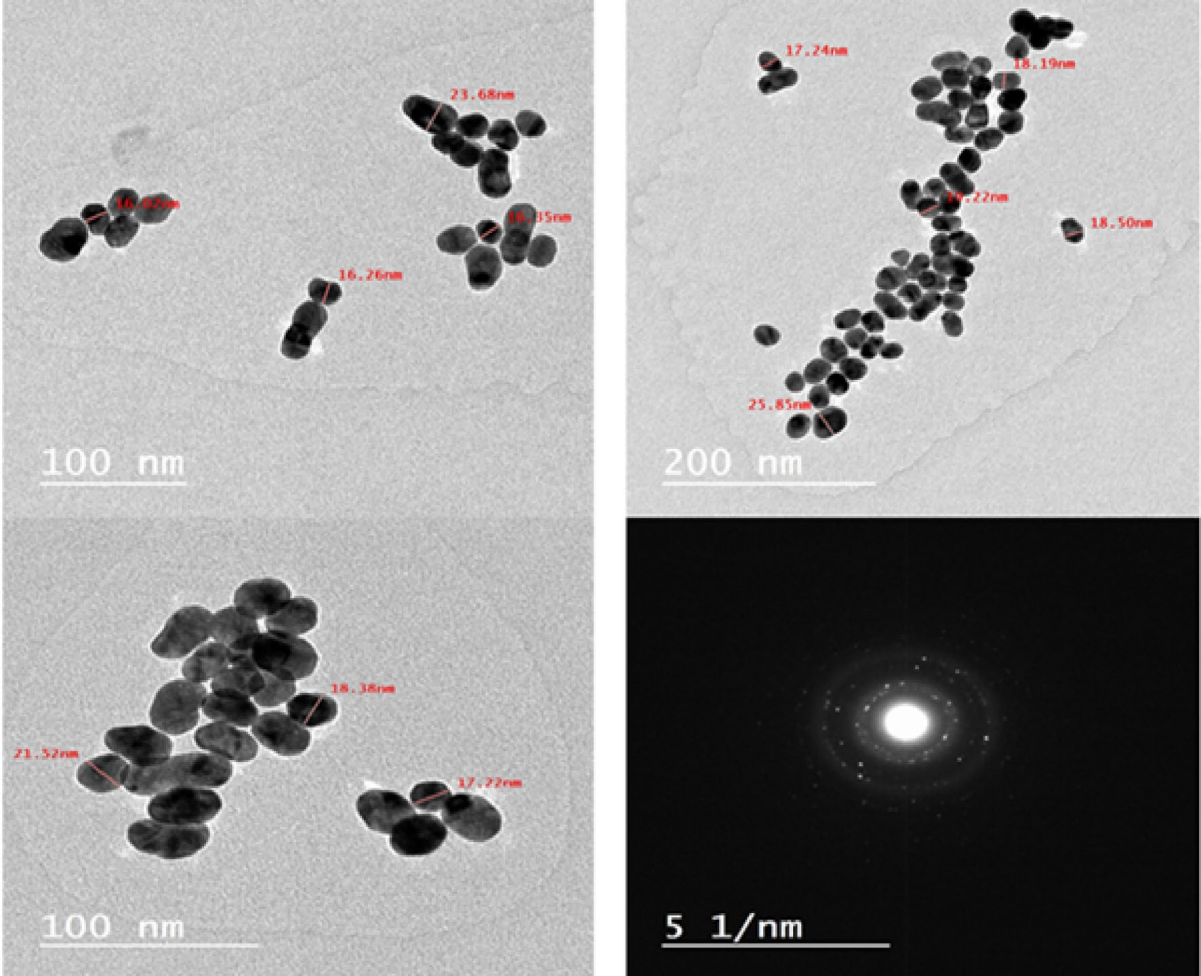
TEM images of prepared Au NPs with different scales (100 and 200 nm) and SAED patterns.

Figure 2 shows typical TEM images of Au NPs (gold nanoparticles) prepared at 30 °C in PEG solutions. Although the shapes of Au NPs are spherical shape structure with an average mean diameter size ranged 20 nm, an apparent size difference is observed between 16 and 25 nm.

**Figure 3:**
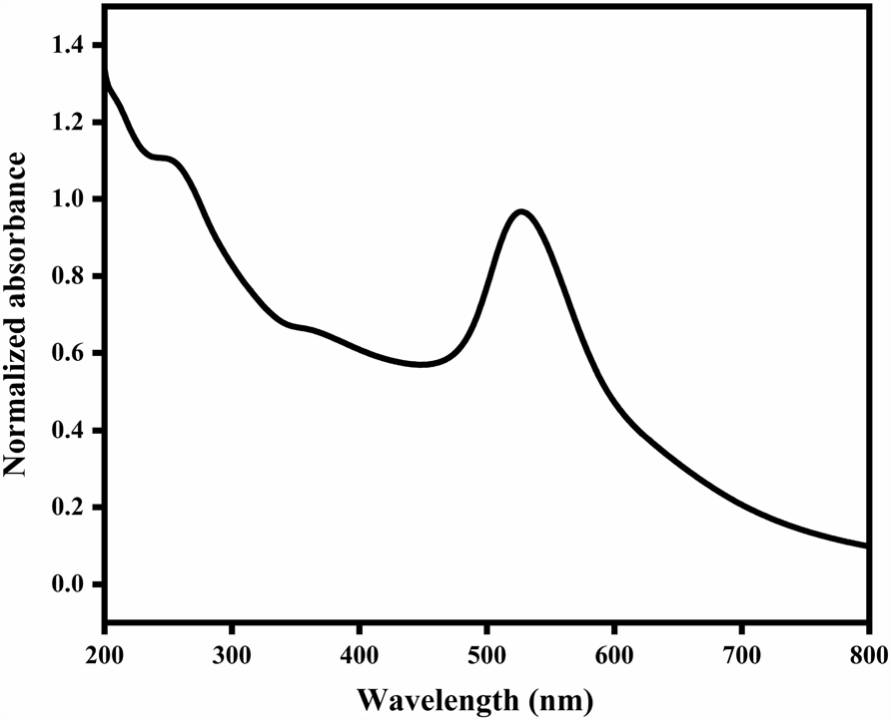
UV–Vis absorption spectra of the prepared Au NPs. The absorbance spectra were normalized at 300 nm.

Figure 3 shows the absorption spectra of the prepared Au NPs prepared in solutions of different sodium citrate concentrations according to the procedure of Naidu et al.^25^. The spectrum is normalized by the absorbance at a wavelength of 300 nm to nullify differences in sample thickness and Au concentration. It was observed that the surface plasmon resonance absorption around λ 520 nm as a peak or shoulder of the Au absorbance fingerprint.

### Analytical sensitivity testing

**Figure 4:**
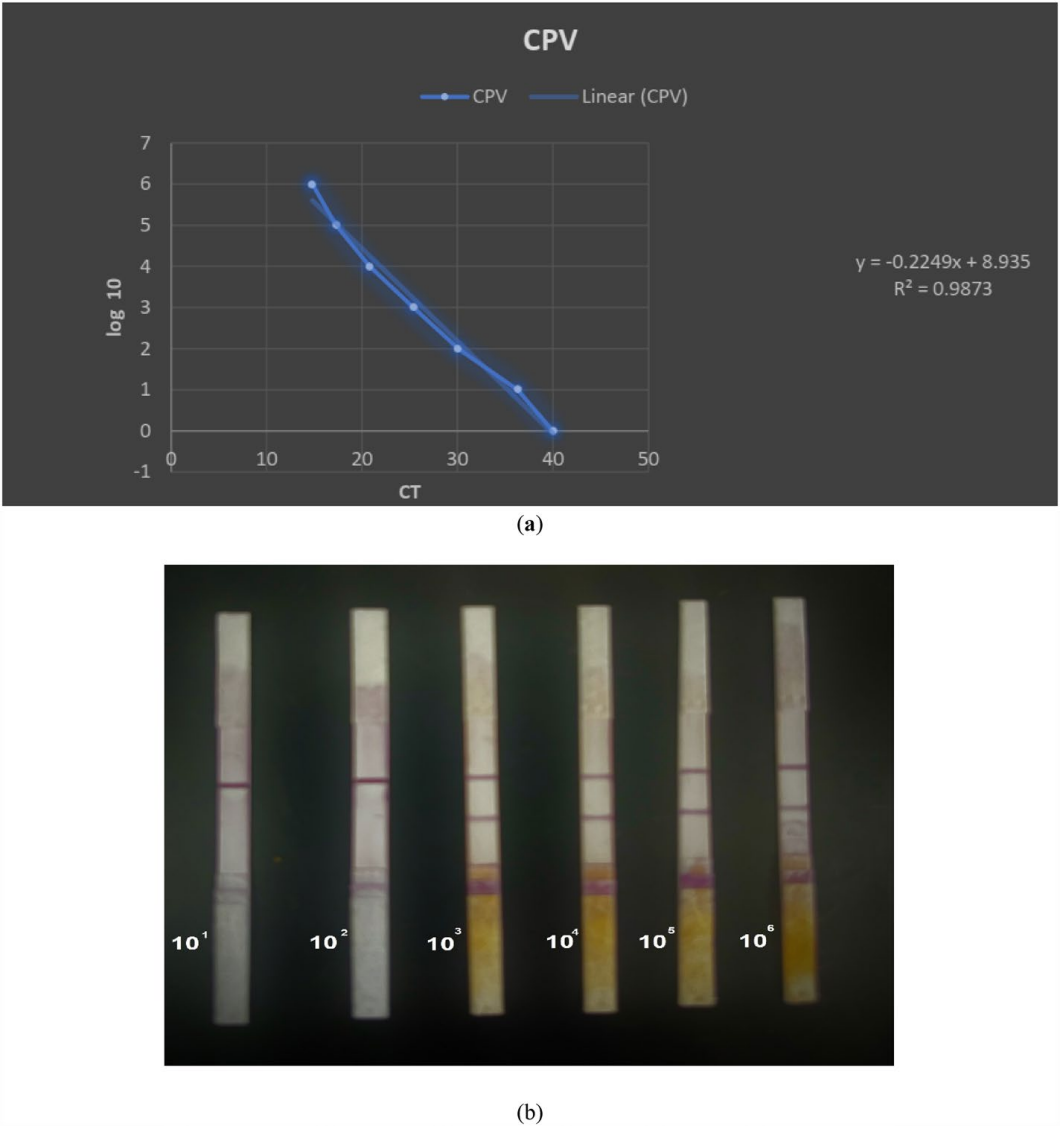
(**a**) Linear range and equation for CPV using qPCR. (**b**) Limit of detection (LOD) of the prepared LFA-CPV antigen using serial dilutions of phosphate buffer saline (PBS) spiked with CPV.

The minimal virus titer (TCID_50_/0.1 mL): The LFA-CPV antigen test could detect a virus titer of 10^3^ TCID_50_/0.1 mL, which was considered a weak or suspected positive result. the virus titer of 10^4^ TCID_50_/0.1 ml was definitely positive, as indicated in Fig. 4. Moreover, the sensitivity testing was confirmed using the real time PCR technique; which showed that the The LFA-CPV antigen test was positive at a Ct value of 20.8 and weakly positive at a Ct value of 25.4 as shown in Table 1.

**Table 1:** The limit of detection (LOD) of CPV antigens by the developed LFA-CPVantigen test, as compared to qPCR.

Method	10^6^	10^5^	10^4^	10^3^	10^2^	10
LFA-CPV antigen	Positive	Positive	Positive	Weakly positive	Negative	Negative
qPCR	Positive (Ct14.8)	Positive (Ct17.3)	Positive (Ct20.8)	Positive (Ct25.4)	Positive (Ct30.1)	Positive (Ct36.3)*

*When the Ct- cycle threshold values of a target gene are be-tween 37 and 40, the sample should be regarded as suspected negative (− ve).

### Analytical specificity testing

The LFA-CPV antigen test indicated positive for CPV antigens and negative for all other tested virus samples, including Canine adenovirus, Canine Parainfluenza, and Canine distmper. Furtehermore, it did not show any interference from interfering substances or tested bacterial strains.

### Evaluation of the developed LFA-CPV antigen test compared with qPCR test

The sensitivity, specificity and accuracy for the developed LFA-CPV antigen test relative to qPCR test were 96.4%, 88.2%, and 95%, respectively, as shown in Table 2.

**Table 2:** The relative sensitivity, specificity and accuracy of the developed LFA-CPV antigen test, as compared with the qPCR test.

A*				
Method	qPCR	Specificity (%)	Accuracy (%)	Total results
LFA-CPV antigen	Results	Positive	Negative	
	Positive	80 (True + ve)	2 (False + ve)	
	Negative	3 (False − ve)	15 (True − ve)	
Total results		83	17	100

B				
Sample	Sensitivity (%)	Specificity (%)	Accuracy (%)	
CPV virus	96.4%	88.2%	95%	

C				
95%CI				
LFA	Lower	Upper		
Negative	0.00	0.35		
Positive	0.94	1.0		
Total mean	0.75	0.90		

*(A) True positive (LFA + PCR+); false positive (LFA + PCR−); true negative (LFA − PCR−); false negative (LFA − PCR+).

(B) The performance of developed LFA-CPV Antigen: sensitivity, specificity and accuracy.

(C) The confidence intervals (95% CI).

## Discussion

Parvovirus infections in dogs pose a significant global challenge. The clinical signs of parvovirus closely mimic those of other enteric diseases, making prompt and early diagnosis essential. In this study, we developed the LFA-CPV Antigen test for rapid diagnosis of Canine Parvovirus infection. The test detects CPV-2 capsid protein VP2 in clinical specimens and live attenuated canine parvovirus vaccine, Monoclonal antibodies (MAbs) specific to recombinant CPV-2 capsid protein VP2 were purchased and conjugated with gold nanoparticles. Additionally, prepared rabbit IgG specific to Canine parvovirus antigens was used to develop the CPV antigen detection LFA^26^.

In this study, a conjugate prepared with 20 nm GNPs demonstrated satisfactory stability and immunological reactivity towards the CPV antigen. current researchs support the use of 20–40 nm gold nanoparticles (GNPs) for conjugate preparation in immunochromatographic (IC) tests^9,27^.

Evaluation of the sensitivity of the developed LFA-CPV antigen test revealed a limit of detection (LOD) of 10^3^ TCID_50_/0.1 ml, producing a weak positive result (Fig. 4). At a virus titer of 10^4^ TCID_50_/0.1 ml, the test yielded a positive result. Correlating the LFA-CPV antigen test results with the gold standard qPCR testing, the LFA-CPV antigen test was positive at a Ct value of 20.8. and weakly positive at a Ct value of 25.4 (Table 1), a level that is marginally higher than the value (3.13 × 10^5^ TCID_50_/ml) reported for the commercial IC based kit to detect CPV in fecal samples. However, this difference in LOD is considered acceptable given the economical benefits of the LFA-CPV antigen test and its ability to detect CPV, especially at early stages of disease when viral loads are typically high^11^, and could be valuble for detection the CPV in the commercial modified live virus (MLV) vaccines which containing attenuated virus (> 10^4^ TCID_50_)^28^.

To validate the specificity of the developed LFA-CPV antigen test, it was evaluated for its ability to detect CPV and differentiate it from other canine viruses. The test accurately identified CPV antigens and produced negative results for all other tested viruses, including Canine adenovirus, Canine Parainfluenza, and Canine distemper. Additionally, the LFA-CPV antigen test demonstrated no cross-reactivity with interfering substances or bacterial strains). In similar studies, the cross-reactivity of the CPV IC test with other pathogens was assessed using standard antigens from canine adenovirus 1 (CAV-1), canine distemper (CD) virus vaccine strain, *Salmonella Typhimurium, Clostridium perfringens* type A, and *Leptospira canicola* (vaccine antigen) on test strips. The specific bacterial and viral agents were procured from the institute repository. To evaluate the stability of the IC strips, assembled strips from a single batch were stored at 4 °C and repeatedly tested with known CPV-positive and negative samples every 15 days for a period of three months^11^.

The performance of the developed LFA-CPV antigen test demonstrated remarkable sensitivity, specificity, and accuracy of 96.4%, 88.2%, and 95%, respectively, when compared to the gold standard qPCR test. Interestingly, another study reported the results of sensitivity, specificity, predictive values and overall accuracy tested by eight lFA- CPV antigen tests compared to qPCR indicated the specificity was excellent for all eight LFA tests (100.0% in all tests), while the sensitivity varied between 32.7 and 49.0%^13^.In another study, a selection of specimens (CPV2a: n = 51; CPV-2b: n = 50; CPV-2c: n = 100) with CPV DNA loads exceeding 10^5^ DNA copies/mg feces, as determined by real-time PCR, were obtained from previous studies. The in-house test exhibited positivity rates of 80.4%, 78.0%, and 77.0% for CPV types 2a, 2b, and 2c, respectively, demonstrating its ability to detect the newly emerged CPV-2c variant. However, given the previously observed sensitivity limitations of the in-house test, negative results should be confirmed using PCR-based methods^29^. The limitations of this study could be addressed as the sensitivity, specificity, and accuracy were assessed using vaccines rather than clinical samples. Determining diagnostic performance using clinical samples is mandatory to show the feasibility of diagnostic tests. Also, samples from experimentally inoculated animals should better serve as the gold standard, user error, non-random sampling, a crucial future direction involves determining the diagnostic performance of the Lateral Flow Assay for Canine Parvovirus (LFA-CPV) using actual clinical samples. This step is imperative to establish the feasibility of the assay in real-world diagnostic scenarios, where the composition of samples may vary widely. It should be noted that both the LFA and qPCR lack the capability to differentiate between live virus and antigen, particularly in the context of a live attenuated vaccine. The study provides information on the overall virus content without the ability to distinguish between live, attenuated virus and viral antigens. To enhance the precision of the study, future investigations could delve deeper into the factors influencing qPCR test results, such as PCR efficiency and the presence of inhibitors. Understanding and addressing these variables will contribute to a more accurate assessment of diagnostic performance.

The study has offered valuable insights into the potential of the Lateral Flow Assay for Canine Parvovirus (LFA-CPV) antigen test. However, it is crucial to acknowledge and address the identified limitations in future research endeavors. This concerted effort will contribute to a more comprehensive and clinically applicable understanding of the developed assay's capabilities. Our findings, once limitations are carefully addressed, will serve as valuable insights into the feasibility of LFA as a point-of-care test for the rapid detection of Canine Parvovirus (CPV) in veterinary practice. Additionally, the LFA could emerge as an alternative method for the preliminary evaluation of CPV vaccines, enhancing its practical applications in the field.

## Conclusion

The Lateral Flow Assay for Canine Parvovirus (LFA-CPV) antigen test, developed in this study, emerges as a promising alternative for evaluating live attenuated Canine Parvovirus (CPV) vaccines in comparison to current laboratory methods, particularly qPCR. Positioned as a point-of-care diagnostic tool for CPV, this assay presents a cost-effective, user-friendly, and rapid on-site solution for both the preliminary evaluation of CPV vaccines.

While demonstrating potential in semi-quantitative analysis, as indicated by its ability to rapidly detect CPV titers, especially those exceeding 10^3^ TCID_50_, further investigation is imperative, particularly for its application with clinical samples. This critical exploration will shed light on its accuracy, reliability, and suitability in the broader context of clinical diagnostics.

### Supplementary Information

Supplementary Information.

## Data Availability

All data generated or analyzed during this study are included in this published article and supplementary file.
