# Supplementary material for: Sensitivity of lateral flow technique for diagnosis of canine parvovirus
Source: Sci Rep. 2024 Mar 1;14:5060. doi: 10.1038/s41598-024-55548-x (PMC10904390; doi:10.1038/s41598-024-55548-x)
Supplement: Supplementary file 1 — Supplementary Information. [file 41598_2024_55548_MOESM1_ESM.docx]

Electronic Supplementary Material for:

**Sensitivity of Lateral flow technique for diagnosis of canine parvovirus.**

Abousenna M.S ^1^, Sayed R.H^1^, Shaimaa A. E ^1^, Shasha F.A ^1^ Sara A.E ^1^ and Darwish D.M ^1^.

^1^ Central Laboratory for Evaluation of Veterinary Biologics, Agricultural Research Center, P.O. Box 131, 11381, Cairo, Egypt.

* Correspondence: [mohamedsamy2020@hotmail.com](mailto:mohamedsamy2020@hotmail.com) : mohamed.abousenna@arc.sci.eg

<https://orcid.org/0000-0003-2202-9544>

**Table S 1 CPV strains in various vaccine batches.**

| **No of batches** | **Type of vaccine** | **Canine parvovirus Strain** |
| --- | --- | --- |
| 15 | Local Monovalent | Strain 39 |
| 16 | Imported Monovalent | Cornell 780916-115 strain |
| 15 | Local Bivalent | Strain 39 |
| 15 | Imported Bivalent | Strain 154 |
| 15 | Imported polyvalent#1 | strain NL-35-D |
| 15 | Imported polyvalent#2 | CPV-2b Bio 12/B |
| 10 | Imported polyvalent#3 | Strain 154 |

**# it is referring to the manufacture**

**Table S 2 Canine viruses strains used for specificity(selectivity) testing of LFA-CPV antigen test.**

| **Virus name** | **Strain** | **Source** |
| --- | --- | --- |
| Canine distmper | **Strain Rockborn** | **Strain bank department at CLEVB** |
| Canine adenovirus | **CAV-1 Abbassia 2002**  **CAV-2 Strain Manhattan** |  |
| Canine Parainfluenza | **Strain Cornell** |  |

**Table S 3 primers and probe of qPCR for CPV**

| **assay** | **Primer/probe** | **Sequence 5′ to 3** | **Polarity** | **Position** | **Amplicon size** |
| --- | --- | --- | --- | --- | --- |
| TaqMan assay | CPV-For | AAACAGGAATTAACTATACTAATATATTTA | + | 4104–4135 | 93 bp |
|  | CPV-Rev | AAATTTGACCATTTGGATAAACT | - | 4176–4198 |  |
|  | Probe | FAM-TGGTCCTTTAACTGCATTAAATAATGTACC- TAMRA | + | 4143–4172 |  |

*The thermal cycle protocol used was the following: activation of iTaq DNA polymerase at 95℃ for 10 min and 40 cycles consisting of denaturation at 95 ℃ for 15 s, primer annealing at 52 ℃ for 30 s and extension at 60 ℃ for 1 min.

* ct value threshold ≥37 indicating a negative outcome.
